# Use of Automated Machine Learning to Detect Undiagnosed Diabetes in US Adults: Development and Validation Study

**DOI:** 10.2196/68260

**Published:** 2025-10-08

**Authors:** Jianxiu Liu, Fred Ssewamala, Ruopeng An, Mengmeng Ji

**Affiliations:** 1 Division of Sports Science and Physical Education, Tsinghua University, Beijing, China; 2 IDG/McGovern Institute for Brain Research, Tsinghua University, Beijing, China; 3 Silver School of Social Work, New York University, New York, NY, United States; 4 McSilver Institute for Poverty Policy and Research, New York University, New York, NY, United States; 5 Constance and Martin Silver Center on Data Science and Social Equity, New York University, New York, NY, United States; 6 Division of Public Health Sciences, Department of Surgery, Washington University in St. Louis, 600 S Taylor Ave, St. Louis, MO, 63110, United States, 1 2179799336

**Keywords:** machine learning, AutoML, self-report, screening, undiagnosed diabetes

## Abstract

**Background:**

Early diagnosis of diabetes is essential for early interventions to slow the progression of dysglycemia and its comorbidities. However, among individuals with diabetes, about 23% were unaware of their condition.

**Objective:**

This study aims to investigate the potential use of automated machine learning (AutoML) models and self-reported data in detecting undiagnosed diabetes among US adults.

**Methods:**

Individual-level data, including biochemical tests for diabetes, demographic characteristics, family history of diabetes, anthropometric measures, dietary intakes, health behaviors, and chronic conditions, were retrieved from the National Health and Nutrition Examination Survey, 1999‐2020. Undiagnosed diabetes was defined as having no prior self-reported diagnosis but meeting diagnostic criteria for elevated hemoglobin A_1c_, fasting plasma glucose, or 2-hour plasma glucose. The H2O AutoML framework, which allows for automated hyperparameter tuning, model selection, and ensemble learning, was used to automate the machine learning workflow. For comparative analysis, 4 traditional machine learning models—logistic regression, support vector machines, random forest, and extreme gradient boosting—were implemented. Model performance was evaluated using the area under the receiver operating characteristic curve.

**Results:**

The study included 11,815 participants aged 20 years and older, comprising 2256 patients with undiagnosed diabetes and 9559 without diabetes. The average age was 59.76 (SD 15.0) years for participants with undiagnosed diabetes and 46.78 (SD 17.2) years for those without diabetes. The AutoML model demonstrated superior performance compared with the 4 traditional machine learning models. The trained AutoML model achieved an area under the receiver operating characteristic curve of 0.909 (95% CI 0.897-0.921) in the test set. The model demonstrated a sensitivity of 70.26%, specificity of 90.46%, positive predictive value of 64.10%, and negative predictive value of 92.61% for identifying undiagnosed diabetes from nondiabetes.

**Conclusions:**

To our knowledge, this study is the first to utilize the AutoML model for detecting undiagnosed diabetes in US adults. The model’s strong performance and applicability to the broader US population make it a promising tool for large-scale diabetes screening efforts.

## Introduction

Diabetes mellitus is the eighth leading cause of death in the United States and contributes to substantial health care costs [1]. In 2021, an estimated 38.4 million Americans of all ages had diabetes, representing 11.6% of the US population [2,3]. Of those with diabetes, 22.8% were unaware of or did not report having diabetes [2,3]. When diabetes is undiagnosed, and consequently hyperglycemia remains unmanaged, severe and irreversible microvascular and macrovascular complications can develop, including diabetic neuropathy, nephropathy, retinopathy, and cardiovascular disease [4-7].

Screening asymptomatic individuals for undiagnosed diabetes enables earlier diagnosis and treatment, ultimately reducing the risk of complications and premature death [8-10]. The latest American Diabetes Association (ADA) and US Preventive Services Task Force guidelines recommend beginning diabetes screenings at the age of 35 years [11,12]. However, diabetes screening guidelines that rely on blood testing are not widely followed. Only 50%‐60% of US adults who met the criteria for screening reported receiving glucose testing within the past 3 years [13,14]. The testing rate was alarmingly low among high-risk groups, including those with low education, low household income, and limited health care access [13,14].

Risk assessment tools for diabetes detection using easily accessible and self-reported data have been proposed, but they have shown low overall accuracy and validity in the general population [15-21]. In recent years, various machine learning algorithms have been used to predict diabetes and have yielded better performance than traditional statistics-based models [22-28]. Few studies have developed machine learning models to detect undiagnosed diabetes in the US population [29,30]. Although 2 studies reported a good overall accuracy of 80%, the quality of a positive prediction by models (ie, precision) was notably low, which could lead to a high number of false positives and unnecessary follow-up testing [29,30].

More recently, there has been growing interest within the health care community in automated machine learning (AutoML), which automates machine learning models’ selection, composition, and parameterization to optimize performance [31-33]. AutoML uses voting and stacking ensemble techniques to combine multiple learning models, often improving classification accuracy more effectively than a single machine learning algorithm. Its automation also reduces human error and bias by impartially exploring a wide range of machine learning models [33]. However, despite its potential, no prior studies have investigated the feasibility and performance of AutoML in screening for undiagnosed diabetes.

This study aimed to investigate the potential use of AutoML and self-reported data in detecting undiagnosed diabetes among US adults in a nationally representative survey. The trained model could aid in detecting undiagnosed diabetes in the general US population, particularly in underserved populations with limited access to blood glucose tests. This study could also promote the adoption of AutoML in diabetes research.

## Methods

### Data Source

Individual-level data were retrieved from the National Health and Nutrition Examination Survey (NHANES), 1999‐2020. NHANES is a nationally representative, repeated cross-sectional study conducted by the National Center for Health Statistics. NHANES adopts a complex, multistage probability sampling design to ensure that the collected data are representative of the noninstitutionalized civilian population in the United States. NHANES includes clinical examinations, selected medical and laboratory tests, and self-reported data. NHANES interviews people in their homes and conducts health examinations in a mobile examination center, including laboratory analysis of blood, urine, and other tissue samples. The detailed study design and methodology of NHANES have been described elsewhere [34,35]. This study followed the CREMLS (Consolidated Reporting Guidelines for Prognostic and Diagnostic Machine Learning Models) [36,37].

### Biochemical Tests for Undiagnosed Diabetes

Following the ADA guidelines [38], diabetes was diagnosed based on elevated levels of hemoglobin A_1c_ (≥6.5%), fasting plasma glucose (≥126 mg/dL), or 2-hour plasma glucose (≥200 mg/dL) during a 75-g oral glucose tolerance test. In this analysis, undiagnosed diabetes was defined as having no prior self-reported diagnosis but meeting any of the diagnosis criteria for elevated hemoglobin A_1c_, fasting plasma glucose level, or oral glucose tolerance test level. The details of the diagnostic method used to define diabetes in this study are provided in Table 1.

**Table 1.** Diagnostic method used to define diabetes in this study.

	Diagnostic method
Diabetic	
Diagnosed diabetes	Answer “Yes” to “Other than during pregnancy, have you ever been told by a doctor or health professional that you have diabetes or sugar diabetes?”
Undiagnosed diabetes	Answer “No” to “Other than during pregnancy, have you ever been told by a doctor or health professional that you have diabetes or sugar diabetes?” AND Any of the following tests meet criteria: HbA_1c_^a^≥6.5% (≥48 mmol/mol). FPG^b^≥126 mg/dL (≥7.0 mmol/L). 2-h PG^c^≥200 mg/dL (≥11.1 mmol/L) during OGTT^d^.
Nondiabetic	
Prediabetes	Does not meet criteria for diabetes diagnosis AND Any of the following tests meet criteria: HbA_1c_ 5.7%‐6.4% (39‐47 mmol/mol) FPG 100‐125 mg/dL (5.6‐6.9 mmol/L) 2-h PG 140‐199 mg/dL (7.8‐11.0 mmol/L)
Normoglycemia	All the following tests meet criteria: HbA_1c_<5.7% (<39 mmol) FPG<100 mg/dL (<5.6 mmol/) 2-h PG<140 mg/dL (<7.8 mmol/L)

a HbA_1c_: hemoglobin A_1c_.

b FPG: fasting plasma glucose.

c PG: plasma glucose.

d OGTT: oral glucose tolerance test.

### Participant Selection

The study utilized data from NHANES 1999‐2020, comprising an initial cohort of 112,502 participants. Participants with self-reported diabetes (n=8657) and those with missing data on self-reported diabetes status (n=9672) were excluded. Individuals aged <20 years (n=43,879) and pregnant females (n=1540) were removed from the cohort. Participants with missing laboratory results for diabetes were further excluded (n=36,939). For inclusion in the nondiabetic group, participants had to meet all 3 test criteria to confirm the absence of diabetes. For the diabetes group, participants were included if at least one test met the diagnostic criteria, even if the other two test results were missing. In total, the study cohort included 11,815 participants and was categorized into 2 groups: 9559 without diabetes and 2256 with undiagnosed diabetes.

### Features

#### Demographic Characteristics

Demographic features included age at the survey, gender (male/female), race/ethnicity (non-Hispanic White, non-Hispanic Black, Mexican American, and other races), educational attainment (lower than 9th-grade education, 9th- to 11th-grade education, high school, some college or associate degree, college or higher), marital status (married, widowed, divorced, separated, never married, living with a partner), and income-to-poverty ratio (ratio of monthly family income to the poverty guidelines).

Family history of diabetes was ascertained from the Medical Conditions Questionnaire: “Including living and deceased, were any of your biological, that is, blood relatives including grandparents, parents, brothers, sisters ever told by a health professional that they had diabetes?”

#### Anthropometric Measures

Participants were weighed in mobile examination centers, wearing only underclothing and an examination gown. Weight was recorded on a digital scale in kilograms. Standing height was measured using a stadiometer with a fixed vertical backboard and an adjustable headpiece. BMI was calculated as measured weight in kilograms divided by height in meters squared. Waist circumference was measured just above the iliac crest using a steel measuring tape.

#### Diet Intake and Behaviors

In NHANES, 24-hour dietary recalls were administered to obtain detailed nutritional intake information from participants. Daily dietary intake (the average of 2 d) of energy (kcal), total fat (g), cholesterol (mg), and total sugars (g) was calculated. The frequency of eating out per week was obtained from the question: “On average, how many times per week do you eat meals prepared in a restaurant?”

#### Health Behaviors

The NHANES physical activity questionnaire included questions about daily and leisure-time activities. The average hours spent in each activity were multiplied by the suggested metabolic equivalent (MET) scores to estimate MET hours per week [39]. Indicators (yes/no) for smoking and drinking were obtained from answers to questions: “In any one year, have you had at least 12 drinks of any type of alcoholic beverage?” and “Have you smoked at least 100 cigarettes in your entire life?”

#### Chronic Conditions

Comorbid conditions were obtained from self-reports to the Medical Conditions Questionnaire, “Have you ever been told by a doctor that you had (medical problem)?” which includes hypertension, rheumatoid arthritis, myocardial infarction, congestive heart failure, coronary heart disease, stroke, liver disease, weak/failing kidneys, and cancer/malignancy of any kind.

### AutoML and Custom Machine Learning Models

The H2O AutoML framework was used in this study to automate the machine learning workflow. The H2O AutoML trains several models, cross-validated by default, by using the following available algorithms: extreme gradient boosting (XGBoost), gradient boosting machine, generalized linear model, distributed random forest, extremely randomized trees, and fully connected deep neural network. H2O AutoML introduces two essential advancements to optimize model performance. First, it fine-tunes base models using a fast random search approach, where hyperparameters are selected from a range of values identified as most impactful. Second, H2O AutoML leverages a sophisticated stacking technique to create two powerful ensemble models: “All models ensemble,” which combines all the base models trained, and “Best of the Family ensemble,” which contains the best-performing models. The stacked ensemble models are designed to leverage the diverse strengths of various algorithms, resulting in a final model that is accurate and generalizable across different datasets. H2O AutoML has built-in functionality for class balancing and handling of missing values. Detailed documentation, as well as directions for algorithms and the implementation of H2O.ai, are available online [40].

We randomly split the dataset into training (70%) and test (30%) sets. AutoML trained diverse base models on the training set, ranking them by cross-validated area under the receiver operating characteristic curve (AUC). Cross-validation was performed using a 5-fold approach, with models iteratively trained on 4 subsets of the training set and validated on the remaining subset. A stacked ensemble (“leader”) was constructed by blending the top-performing base models via a meta learner. This stacked ensemble model was exported and applied to our independent holdout test set to generate class-probability predictions. The 95% CIs for each AUC were derived from 1000 bootstrap resamples of the test set. Confusion matrices were constructed to calculate sensitivity, specificity, positive predictive value (PPV), and negative predictive value (NPV) using the classification threshold that maximized the *F*_1_-score that yields the highest harmonic mean of precision and recall on the test set. We extracted feature importance from each base learner and computed a weighted aggregate in the H2O leader model. For tree-based models, feature importance was determined by the frequency of each feature used for splitting and the overall reduction in squared error. For non–tree-based models, importance was based on coefficient magnitudes.

In addition to the AutoML model, we conducted a comparative analysis using 4 traditional machine learning models—logistic regression, support vector machines, random forest, and XGBoost, with Synthetic Minority Over-sampling Technique applied to address data imbalance during training [41,42].

The clinical guidelines generally recommend confirming an elevated test with a secondary measurement for the diagnosis of diabetes [38,43]. In the main analyses, diabetes was diagnosed based on a single elevated test result. In additional analyses, the diagnosis of diabetes was confirmed by at least 2 elevated tests recommended by the ADA guidelines. We also evaluated the performance of a 3-class prediction model for diabetes within the AutoML framework, using classification schemes that distinguished between normoglycemia, prediabetes, and undiagnosed diabetes.

Summary statistics for participant characteristics, stratified by diabetes status, were calculated. Categorical variables were compared using *χ*^2^ tests, and continuous variables were evaluated using independent samples *t* tests. Missing data were imputed using mean values for continuous variables and mode for categorical variables. We used STATA 18 (StataCorp LLC) for data preparation and Python version 3.10.12 (Python Software Foundation) to implement the H2O AutoML (H2O.ai, Inc) and custom machine learning models (version 3.46.0.6).

### Ethical Considerations

This study used publicly available, deidentified NHANES data. In accordance with the US Department of Health and Human Services (Title 45 of the Code of Federal Regulations; §46.104 (d), section 4) [44], analyses of publicly available, deidentified data are not considered human subjects research and therefore do not require review by the Washington University Institutional Review Board.

## Results

**Figure 1.**
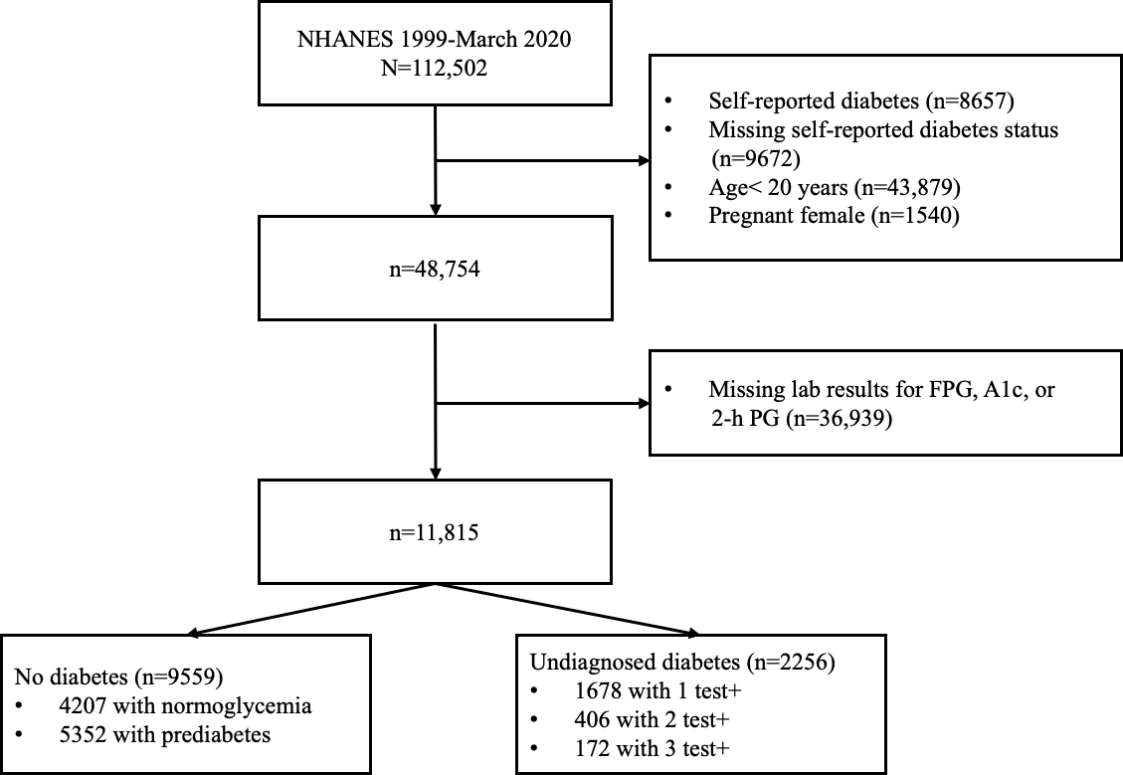
Flow diagram of participant selection. 2-h PG: 2-hour plasma glucose; A_1c_: hemoglobin A_1c_; FPG: fasting plasma glucose; NHANES: National Health and Nutrition Examination Survey.

In total, the study cohort included 11,815 participants, with 9559 participants without diabetes and 2256 with undiagnosed diabetes. The characteristics of the study cohort are summarized in Table 2. The average ages were 59.76 (SD 15.0) years for those with undiagnosed diabetes and 46.78 (SD 17.2) years for those without. The diabetes group had a higher proportion of males, lower levels of education, and a greater likelihood of having a relative with diabetes. Additionally, patients with undiagnosed diabetes had higher BMI, larger waist circumference, and a higher prevalence of chronic conditions. The flow diagram of participant selection is presented in Figure 1.

**Table 2.** Cohort characteristics by diabetes status.

	No diabetes (n=9559)	Undiagnosed diabetes (n=2256)	*P* value
Laboratory tests, mean (SD)			
Glycohemoglobin (%)	5.41 (0.38)	6.88 (1.60)	<.001
Fasting glucose plasma (mg/dL)	98.18 (9.46)	141.38 (46.79)	<.001
Oral glucose tolerance test (mg/dL)	109.53 (31.95)	229.24 (75.68)	<.001
Demographic characteristics			
Age (years), mean (SD)	46.78 (17.19)	59.76 (14.98)	<.001
Gender, n (%)			.005
Male	4711 (49.28)	1186 (52.57)	
Female	4848 (50.72)	1070 (47.43)	
Race/ethnicity, n (%)			<.001
Non-Hispanic White	4381 (45.83)	835 (37.01)	
Non-Hispanic Black	1750 (18.31)	504 (22.34)	
Hispanic	2459 (25.72)	698 (30.94)	
Other	969 (10.14)	219 (9.71)	
Education, n (%)			<.001
≤9 grade	881 (9.22)	434 (19.28)	
9th-11th grade	1301 (13.62)	391 (17.37)	
High school	2127 (22.27)	554 (24.61)	
Some college	2801 (29.32)	543 (24.12)	
College or above	2443 (25.57)	329 (14.62)	
Marital status, n (%)			<.001
Married	5016 (52.47)	1248 (55.54)	
Widowed	568 (5.94)	390 (17.36)	
Divorced	950 (9.94)	251 (11.17)	
Separated	330 (3.45)	72 (3.20)	
Never married	1843 (19.28)	192 (8.54)	
Living with partner	852 (8.91)	94 (4.18)	
Income-to-poverty ratio, mean (SD)	2.60 (1.64)	2.29 (1.53)	<.001
Family history of diabetes, n (%)	3394 (36.16)	1072 (48.79)	<.001
Anthropometric measures, mean (SD)			
Height (cm)	167.89 (10.00)	165.84 (10.24)	<.001
Weight (kg)	80.12 (20.69)	88.19 (23.32)	<.001
BMI (kg/m^2^)	28.33 (6.53)	31.94 (7.43)	<.001
Waist circumference (cm)	96.92 (15.32)	107.5 (15.69)	<.001
Diet intake and eating behavior, mean (SD)			
Daily total energy (kcal)	2090.13 (837.69)	1912.17 (851.19)	<.001
Daily total fat (g)	78.27 (38.36)	72.29 (39.76)	<.001
Daily total sugars (g)	113.59 (65.37)	104.57 (68.14)	<.001
Daily total cholesterol (mg)	287.05 (193.07)	285.82 (201.17)	.80
Times of dining out per week	3.43 (3.84)	2.60 (3.43)	<.001
Health behaviors			
Physical activity (MET^a^-h/wk), mean (SD)	2.36 (3.69)	1.62 (2.81)	<.001
Smoking, n (%)	4200 (43.97)	1031 (48.91)	<.001
Drinking, n (%)	6758 (74.26)	1262 (67.52)	<.001
Self-reported chronic conditions, n (%)			
Hypertension	2778 (29.10)	1188 (52.85)	<.001
Rheumatoid arthritis	2206 (23.12)	778 (34.55)	<.001
Myocardial infarction	283 (2.96)	152 (6.76)	<.001
Congestive heart failure	182 (1.91)	112 (4.99)	<.001
Coronary heart disease	261 (2.74)	145 (6.47)	<.001
Stroke	231 (2.42)	127 (5.64)	<.001
Liver disease	295 (3.09)	117 (5.19)	<.001
Weak/failing kidneys	199 (2.08)	64 (2.84)	.03
Cancer	760 (7.95)	257 (11.40)	<.001

a MET: metabolic equivalent.

**Figure 2.**
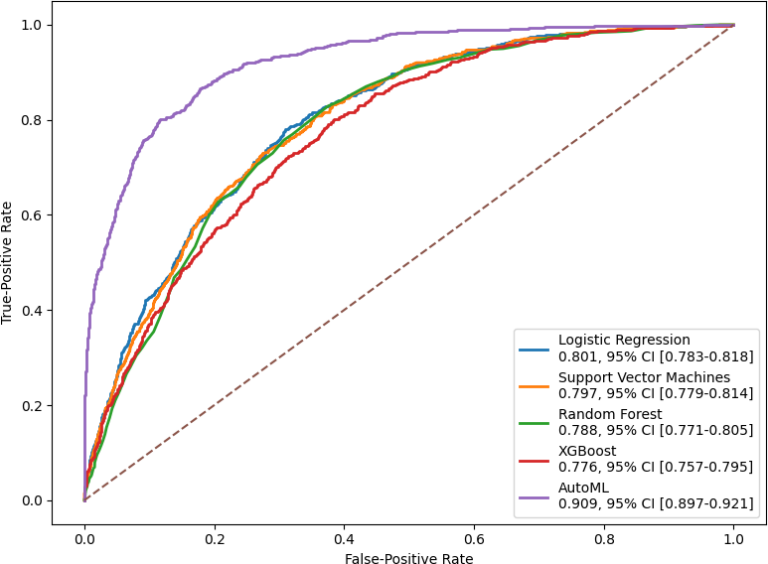
Performance of AutoML model and custom machine learning models in detecting undiagnosed diabetes on the test set. AutoML: automated machine learning; XGBoost: extreme gradient boosting.

The performance of the AutoML model and traditional machine learning models is summarized in Figure 2. The AutoML model demonstrated superior performance compared to the 4 traditional machine learning models—logistic regression, support vector machines, random forest, and XGBoost. The trained AutoML model achieved an AUC of 0.909 (95% CI 0.897-0.921) and an accuracy of 86.5% in the test set. The model demonstrated a sensitivity of 70.26%, specificity of 90.46%, PPV of 64.10%, and NPV of 92.61% for identifying undiagnosed diabetes from nondiabetes (Table 3). The model summary and details were provided in Multimedia Appendix 1.

**Table 3.** Confusion matrix for the classification of undiagnosed diabetes using the AutoML model.^a^

	Predicted label, n (%)	
	No diabetes	Undiagnosed diabetes
True label		
No diabetes	2558 (73)	270 (8)
Undiagnosed diabetes	204 (6)	482 (14)

a Note: The cutoff threshold was 0.248718, optimized for *F*_1_-score that maximized the harmonic mean of precision and recall. The matrix shows the distribution of true labels against predicted labels. The cell values indicate the number of instances (absolute counts) and their corresponding percentages of the total in the test data. Sensitivity (482/686, 70.26%), specificity (2558/2828, 90.45%), positive predictive value (482/752, 64.10%), and negative predictive value (2558/2762, 92.61%) are derived from the matrix to assess model performance.

The top 5 features are age, waist circumference, daily total sugar intake, income, and BMI, together accounting for 50% of total model importance. Comorbidities, except hypertension, contributed minimally relative to demographic and behavioral factors. Excluding comorbidities (except hypertension) resulted in comparable model performance, with an AUC of 0.830 and an accuracy of 85.1%.

Additional analysis results are summarized in Table 4. The model using diabetes diagnosis criteria with a confirmatory test achieved a testing accuracy of 98.0% and 89.7% with an AUC of 0.823. However, precision (ie, PPV) and recall (ie, sensitivity) were suboptimal due to the small number of patients meeting the diabetes criteria with ≥2 tests. The model demonstrated a sensitivity of 44.10%, specificity of 92.22%, PPV of 24.23%, and NPV of 96.69% for identifying undiagnosed diabetes from nondiabetes. The performance of the multiclass prediction model was poor, with an overall accuracy of 58.9% for using diagnosis criteria with ≥1 test and 67.1% for using diagnosis criteria with ≥2 tests.

**Table 4.** Additional models for alternative diabetes diagnosis criteria with the second confirmative test and multiclass prediction including prediabetes.

	Undiagnosed diabetes (≥2 test) versus no diabetes^a^		Undiagnosed diabetes (≥1 test) versus prediabetes versus normoglycemia		Undiagnosed diabetes (≥2 test) versus prediabetes versus normoglycemia	
	Train	Test	Train	Test	Train	Test
Accuracy (%)	98.0	89.7	66.8	59.0	68.6	67.1
Overall AUC^b^	0.993	0.823	0.627	0.557	0.588	0.557
AUC (normal versus rest)	—^c^	—	0.502	0.445	0.316	0.307
AUC (prediabetes versus rest)	—	—	0.814	0.652	0.792	0.715
AUC (diabetes versus rest)	—	—	0.415	0.545	0.555	0.673

a “No diabetes” group includes normoglycemia and prediabetes.

b AUC: area under the receiver operating characteristic curve.

c Not applicable.

## Discussion

To our knowledge, this study is the first to utilize the AutoML model for detecting undiagnosed diabetes in US adults. The best-performing model achieved an AUC of 0.91 and an accuracy of 86.5% in the test set. National surveillance shows that nearly half of those with undiagnosed diabetes have hypertension, lipid abnormalities, or cardiovascular and chronic kidney diseases [45-48]. Delayed diagnosis of diabetes hinders the opportunities for early intervention to slow the progression of dysglycemia and its comorbidities. Our model was trained and tested using a substantial and diverse dataset comprising nationally representative survey data. The model’s high accuracy and applicability to the broader US population make it a promising tool for large-scale diabetes screening efforts.

The feature importance ranking of the best-performing model highlights waist circumference, BMI, and dietary variables as key predictors, underscoring their strong links to metabolic health, insulin resistance, and dietary habits that influence diabetes risk. Lifestyle factors such as drinking frequency and physical activity also emerged as significant contributors, whereas self-reported comorbidities played a smaller role once anthropometric and behavioral measures were included. These findings align with epidemiological studies and improve the model’s interpretability, providing actionable insights to prioritize targeted interventions for modifiable risk factors [49].

Machine learning has advanced clinical research but faces adoption barriers like data access, imbalances, and reliance on data science expertise for deployment [50,51]. AutoML reduces the need for machine learning expertise, enabling clinicians to use advanced technologies without programming skills and integrating them into research and clinical practice [52-55]. Despite being promising, few studies have explored the application of AutoML for diabetes diagnosis [56].

Previous studies have compared traditional machine learning models with conventional statistical models for identifying undiagnosed diabetes, demonstrating that machine learning models outperform statistical models [29,30]. These studies reported AUC values between 0.73 and 0.81, consistent with the performance of traditional models in this study. However, the reported low PPVs highlighted the limited ability of these models to accurately identify undiagnosed diabetes cases [30]. This study showed that AutoML models are superior and outperformed traditional machine learning models in detecting undiagnosed diabetes. Similarly, one study has reported that the AutoML model outperformed both individual and ensemble models in identifying patients with diabetes using electronic medical records data [57]. These findings suggest that AutoML provides a more accessible and efficient approach, eliminating the need for manual optimization while delivering superior performance.

Nonetheless, several issues with the AutoML model in diabetes screening should be noted. When applying more stringent diabetes diagnostic criteria, the accuracy reached 90%; however, precision and recall were low, likely due to the limited number of samples that met the ≥2 test criteria. H2O AutoML provides significant advantages, including its built-in class balancing functionality, which automates the handling of moderate class imbalance without requiring external implementations. Using random sampling to upsample minority classes or downsample majority classes, it effectively manages datasets with moderate imbalance, such as the 4:1 ratio under the 1+ test criterion for diabetes diagnosis in this study. However, with the stricter 2+ test criterion, the imbalance ratio rose to 24:1, likely exceeding H2O AutoML’s ability to mitigate the imbalance. This severe imbalance impacted the model’s ability to accurately distinguish undiagnosed diabetes from nondiabetes in unseen data, reducing precision and recall. For such highly imbalanced datasets, combining AutoML with other data resampling methods, such as Synthetic Minority Over-sampling Technique, could better improve model performance [58].

In addition, the model’s performance in multiclass classification of no diabetes, prediabetes, and undiagnosed diabetes was notably poor. Prediabetes is an intermediate stage between normal glycemia and diabetes and is highly prevalent [59]. Clinically, prediabetes and diabetes, as the continuum of dysglycemia, share many overlapping risk factors, such as insulin resistance and elevated glucose levels [60], making their differentiation challenging. The subtle metabolic differences between these conditions may not have been adequately captured by the included self-reported data. Future efforts should focus on incorporating additional features and refining the model architecture to enhance accuracy and improve its ability to identify prediabetes.

A major strength of this model is its use of NHANES data, which is nationally representative of the US population, enhancing the generalizability of the findings. The model incorporates comprehensive self-reported data, including nutritional information, for predicting undiagnosed diabetes. The application of AutoML in this study represents the first use of this approach in diabetes research, providing a foundation for further development and validation of similar models. However, the widespread adoption of our AutoML in health care requires further development and validation. False positives can lead to unnecessary tests, higher costs, and patient anxiety, while false negatives may delay treatment and worsen outcomes. Using AutoML models in real-world clinical settings requires meticulous threshold optimization and validation to achieve an appropriate balance between precision and recall. These models should be rigorously evaluated for fairness and equity, ensuring that performance does not vary significantly across demographic groups [61,62]. Practical barriers to implementing AutoML in clinical practice also include integration with electronic health record systems and the lack of trust in “black-box” models due to their opacity [31,61-63].

Several limitations should be noted. First, the definition of undiagnosed diabetes in the base model was based on a single elevated measurement, which may not fully capture the condition. However, the model still showed utility as a screening tool. Second, the model demonstrated poor performance in multiclass prediction, including prediabetes, indicating that additional feature refinement and model adjustments may be necessary to improve accuracy in 3-class predictions. Third, known diabetes diagnoses rely on self-reports, which may introduce potential recall bias and misclassification. This could lead to mislabeling cases and diminishing model performance. Although most published public health studies still rely on self-reports—hence making our study not an unusual one in using self-reports—future studies should aim to validate self-reported data against medical records where feasible to minimize errors and increase reliability of the observations. Finally, the generalizability of this model may be limited to US populations and may not extend to non-US populations.

This study demonstrates the potential of AutoML in detecting undiagnosed diabetes using self-reported and easily accessible data. Although challenges remain in accurately classifying multiple categories, including prediabetes, the model shows promise as a tool for large-scale diabetes screening. Further refinement and validation are required to improve its applicability across diverse populations.

## Supplementary material

10.2196/68260 Multimedia Appendix 1 Model summary for stacked ensemble.

## Abbreviations

ADA: American Diabetes Association
AUC: area under the receiver operating characteristic curve
AutoML: automated machine learning
CREMLS: Consolidated Reporting Guidelines for Prognostic and Diagnostic Machine Learning Models
MET: metabolic equivalent
NHANES: National Health and Nutrition Examination Survey
NPV: negative predictive value
PPV: positive predictive value
XGBoost: extreme gradient boosting
