# Supplementary material for: Use of Automated Machine Learning to Detect Undiagnosed Diabetes in US Adults: Development and Validation Study
Source: JMIR AI. 2025 Oct 8;4:e68260. doi: 10.2196/68260 (PMC12532270; doi:10.2196/68260)
Supplement: Multimedia Appendix 1 [file ai-v4-e68260-s001.docx]

**Appendix 2:** Model Summary for Stacked Ensemble.

| Key | Value |
| --- | --- |
| Model ID | StackedEnsemble_AllModels_4_AutoML_2_20240825_25524 |
| Stacking strategy | Cross validation |
| Number of base models (used / total) | 20/107 |
| # GBM base models (used / total) | 3/37 |
| #XGBoost base models (used / total) | 14/58 |
| # DRF base models (used / total) | 1/2 |
| # GLM base models (used / total) | 0/1 |
| # DeepLearning base models (used / total) | 2/9 |
| Metalearner algorithm | GLM |
| Metalearner fold assignment scheme | Random |
| Metalearner nfolds | 5 |
| Metalearner fold_column | None |
| Custom metalearner hyperparameters | None |

Note: The model MOJO file has been uploaded to a public GitHub repository for reproducibility. The repository is available at: https://github.com/Mengmeng1993/H2OautoML_undiagnosed_Diabetes.
